# Prognostic Significance of Macrophage Phenotypes in Peri-Tumoral Normal Tissue of Early-Stage Breast Cancer

**DOI:** 10.3390/cells14110828

**Published:** 2025-06-03

**Authors:** Marcel Hirschmann, Sören Schnellhardt, Matthias Rübner, Sarah Segelhorst, Oliver Ott, Ramona Erber, Christoph Daniel, Maike Büttner-Herold, Paul Gass, Rainer Fietkau, Luitpold Distel

**Affiliations:** 1 Department of Radiation Oncology, Universitätsklinikum Erlangen, Friedrich-Alexander-Universität Erlangen-Nürnberg (FAU), Universitätsstraße 27, 91054 Erlangen, Germany; 2 Comprehensive Cancer Center Erlangen-EMN (CCC ER-EMN), 91054 Erlangen, Germany; 3 Department of Radiotherapy and Radiation Oncology, Saarland University Medical Center, 66421 Homburg, Germany; 4 Frauenklinik, Comprehensive Cancer Center Erlangen-Europäische Metropolregion Nürnberg (EMN), Uniklinikum Erlangen, Friedrich-Alexander-Universität Erlangen-Nürnberg (FAU), Östliche Stadtmauerstraße 30, 91054 Erlangen, Germany; 5 Institute of Pathology, University Regensburg, Franz-Josef-Strauß-Allee 11, 93053 Regensburg, Germany; 6 Department of Nephropathology, Institute of Pathology, Universitätsklinikum Erlangen, Friedrich-Alexander-Universität Erlangen-Nürnberg (FAU), 91054 Erlangen, Germany; 7 Klinikum Chemnitz gGmbH, Medizincampus Chemnitz der Technischen Universität Dresden, 09116 Chemnitz, Germany

**Keywords:** early-stage breast cancer, normal tissue, M1, M2, macrophages, tumor-infiltrating inflammatory cells, microenvironment, radiotherapy, partial breast irradiation

## Abstract

In recent years, tumor-infiltrating inflammatory cells within the tumor microenvironment have been extensively studied. However, much less is known about inflammatory cells in the normal tissue surrounding tumors. In this study, we assess the prognostic significance of tumor-associated macrophages (TAMs) in relation to disease-free survival (DFS) in patients with early-stage breast cancer. Our cohorts included patients from the APBI and BBCC trials, with eligible tumors being small in size and showing no signs of metastasis. We analyzed eight distinct inflammatory cell types in the normal tissue surrounding tumors, with a particular focus on the various macrophage subsets. There were clear differences in the frequencies of the different inflammatory cells, with a higher abundance of cells being found in the intraepithelial compartment compared to the stromal compartment. Notably, we found that M2-type macrophages located in the stromal compartment of tumor distant normal tissue exhibited a positive prognostic impact, in contrast to the M2-type macrophages found within the tumor itself. In the normal tissue surrounding tumors, there are surprisingly clear prognostic predictions for DFS. Normal tissue surrounding breast cancer tumors is clearly influenced by the tumor and could also influence the tumor in terms of growth and metastasis. Tumor-influenced inflammatory cells in the surrounding normal tissue could prevent the immune system from acting against the tumor and promote tumor growth through inflammation.

## 1. Introduction

Breast cancer is the most diagnosed malignancy worldwide, with approximately 2.3 million new cases reported, making up for about 11.8% of all cancers diagnosed in 2022 [1]. Breast cancer remains the second leading cause of cancer-related death in women, despite advances in early detection and treatment that have helped reduce overall mortality [2,3]. Metastatic disease can complicate treatment and reduce the likelihood of cure by spreading to distant organs such as the bones, liver, lungs, and brain [4]. Consequently, metastatic breast cancer patients typically have a poor prognosis [5]. However, early-stage breast cancer is potentially curable, and treatment strategies have improved over time [6]. The focus has shifted to optimizing the intensity of therapy to avoid overtreatment [7].

There are several strategies for treating early-stage breast cancer. Locoregional treatment generally involves surgery and radiation therapy at the site of the tumor [8]. Systemic therapy is designed to target the specific characteristics of cancer cells. These include the presence of steroid hormone receptors (HRs) and human epidermal growth factor receptor 2 (HER2). Breast cancer cells can be classified based on the receptors they express. Tumor cells that express estrogen receptors (ERs), progesterone receptors (PRs), or both are classified as ER-positive (ER+), PR-positive (PR+), or ER/PR-positive (ER+/PR+) breast cancer [9]. New therapeutic approaches focused on harnessing the potential of the immune system have emerged in recent years [10].

Tumor-infiltrating inflammatory cells, particularly tumor-infiltrating lymphocytes (TILs), have been shown to be significant predictors of response to neoadjuvant chemotherapy [11]. Thus, targeted therapies, such as immunotherapy, are becoming an increasingly valuable tool in the treatment of breast cancer [12]. In particular, the tumor microenvironment (TME) and its stromal cells have attracted attention due to their molecular alterations and aberrant signaling pathways [13]. A critical component of the TME are tumor-associated macrophages (TAMs), which can polarize into two major subtypes: M1-type and M2-type macrophages [14]. Macrophages of the M1-type have a pro-inflammatory cytotoxic phenotype, whereas macrophages of the M2-type have an immunosuppressive phenotype and are involved in tissue remodeling and angiogenesis [14].

Our understanding of tumors has changed dramatically in recent years. In the past, it was assumed that there was a mass of tumor cells that needed to be treated. Over time, it has been understood that the connective tissue within the tumor and its components have an important function [15]. Even more important is the role of tumor-infiltrating inflammatory cells and tumor-associated connective tissue [16]. However, what is still poorly understood is the exact spatial extent of the TME, whether normal tissue surrounding the tumor is affected by it, and whether it may also have an influence on the tumor and its growth and metastasis. This led us to analyze the normal tissue surrounding the tumor in two different cohorts of early-stage breast cancer patients with the goal of identifying the types of inflammatory cells present, particularly macrophages, and investigating their prognostic significance in the context of early-stage breast cancer.

## 2. Materials and Methods

### 2.1. Clinical Characteristics of the APBI Study

Patients were recruited between 2000 and 2005 as part of the German–Austrian Accelerated Partial Breast Irradiation (APBI) Phase II trial at the Universitätsklinikum Erlangen. A total of 146 patients with early-stage breast cancer were enrolled in the subgroup of the study analyzed in this research. Inclusion criteria required participants to be at least 35 years of age with a histopathological confirmed diagnosis of invasive breast carcinoma of any subtype. The mean age of the patients was 59.1 years. Eligible tumors had to be unifocal, unicentric, and no larger than 3 cm in diameter. Resection margins had to be tumor-free with a minimum clearance of 2 mm in all directions [17].

All cancers were hormone receptor-positive, either for estrogen (ER+/PR−), progesterone (ER−/PR+), or both (ER+/PR+). Molecular subtyping identified 97 cases as Luminal A and 44 cases as Luminal B. None of the patients had lymphatic or vascular invasion, distant metastases, or axillary lymph node involvement [17].

All patients underwent breast-conserving surgery followed by either interstitial multi-catheter pulsed-dose-rate (PDR) or high-dose-rate (HDR) brachytherapy. Hormone therapy was administered to 131 (89.7%) patients, while 10 (6.8%) patients received chemotherapy. The DFS rate for this subgroup of the APBI trial was 85% over 12 years, while breast cancer-specific survival was 99% (Table 1) [17].

Prior to participation in the trial, written informed consent was obtained from all patients for tissue sampling and clinical data collection. The Ethics Committee of the Friedrich-Alexander-Universität Erlangen-Nürnberg (FAU) approved the use of formalin-fixed paraffin-embedded samples from the Institute of Pathology’s archive and waived the requirement for additional consent for accessing the archived material [17]. Samples of 129 patients out of the 146 patients initially recruited were used in this study, since some TMA samples were left out due to losses during the staining process or bad quality of single TMA spots.

### 2.2. Clinical Characteristics of the BBCC Study

From 2002 to 2007, a total of 1538 patients were enrolled in the Bavarian Breast Cancer Cases and Controls (BBCC) study [18]. Tissue microarrays were constructed for 880 of those patients. Data on disease-free survival (DFS), metastasis, and recurrence were collected for 10 years after the start of data collection. For our study, we excluded patients with a previous breast cancer diagnosis, as well as patients with metastases at diagnosis. Patients with incomplete datasets or damaged TMAs were also excluded, leaving a total of 501 patients in our study (Table 1). All patients provided written informed consent for tissue sampling and clinical data collection prior to participating in the trial. The Ethics Committee of Friedrich-Alexander University Erlangen-Nürnberg (FAU) approved the use of formalin-fixed paraffin-embedded samples from the Institute of Pathology’s archive and waived the need for additional consent to access the archived material.

### 2.3. Tissue Microarray (TMA) Construction

Formalin-fixed paraffin-embedded tumor specimens were used to construct tissue microarrays. In the APBI study, normal tissue very close to the tumor within five millimeters of the tumor was classified as tumor-near, and more distant normal tissue was classified as tumor-distant, which was generally no more than ten millimeters away from the tumor. In the BBCC study, tissue located between 3 mm and 10 mm outside the tumor was classified as normal tissue [19]. The normal tissues were identified and marked by a pathologist on a Hematoxylin and Eosin (HE)-stained section of the whole tissue block. This slide was then scanned, and the marked area was digitally transferred to an image of the paraffin block. This transfer was reviewed by a pathologist and then the spots were automatically punched out and inserted into a paraffin recipient block. The normal tissue spots were two millimeters in diameter.

### 2.4. Tissue Staining

Tissue two-color immunohistochemistry staining was performed. Sections were deparaffinized and rehydrated, and antigen retrieval was performed in a steam cooker for 5 min. The antibodies used were as follows:

○ CD68-specific monoclonal mouse antibody (1:1500. PG-M1, Dako GA613, Agilent Technologies, Santa Clara, CA, USA);
○ CD206-specific monoclonal mouse antibody (1:100, Novus biologicals NBP2-52927, Abingdon, UK);
○ MPO-specific polyclonal rabbit antibody (1:200, Abcam ab9535, Cambridge, UK);
○ CD163-specific monoclonal mouse antibody (1:3000, clone 10D6, Novocastra NCL-L-CD163, Leica, Nussloch, Germany);
○ FoxP3-specific monoclonal mouse antibody (1:100, clone 236A/E7, Abcam ab20034, Cambridge, UK);
○ CD8-specific monoclonal mouse antibody (1:50, clone C8/144B, Agilent M7103, Santa Clara, CA, USA) [20];
○ Monoclonal mouse anti-human CD45RO antibody (DAKO Denmark A/S, Glostrup, Denmark);
○ CD1a mouse monoclonal, (clone CD1a007, Abcam ab708, Cambridge, UK);
○ CD20 (1:2000, mouse monoclonal, clone L26, Dako M0755, Agilent Technologies, Santa Clara, CA, USA);
○ Clonal mouse anti-human CD45RO antibody (DAKO Agilent Technologies, Santa Clara, CA, USA);
○ CD4 (Clone C8/144B, Leica NCL-CD4-1F6, Nussloch, Germany) [17].

For double-staining, the first antibody was applied overnight and detected using a Polymer-Kit (Fa. Zytomed POLAP-100, Zytomed, Langenzersdorf, Austria) and Fast Red chromogen. Then, the second antibody was added for 60 min, and detection was performed with the Polymer-Kit and Fast Blue chromogen. We previously published the methodology of staining and the cell density results of CD1a+ dendritic cells, CD20+ B cells, CD45RO memory T cells, and CD4 helper T cells in the APBI cohort [17]. No isotype controls were carried out. Stained samples were scanned using a whole-slide scanner (ZEISS Axio Imager 2, Zeiss, Oberkochen, Germany) at 0.11 µm/pixel.

Four-color immunofluorescence staining was performed with four antibodies that were directly fluorescently labeled. B cells were stained with AlexaFluor 488-labeled anti-CD21 (Abcam, Cambridge, UK, rabbit monoclonal, clone [EP3093] (ab75985)); activated cells were stained with AlexaFluor 555-labeled anti-CD27 (Abcam, rabbit monoclonal, clone EPR8569 (ab282112)); B cells were stained with AlexaFluor 647-labeled CD19 (Abcam, rabbit monoclonal, clone EPR5906 (ab196515)); and memory B cells were stained with AlexaFluor 750-labeled CD20 (Dako, mouse monoclonal, clone L26, M0755). Cell nuclei were stained with 4′,6-diamidino-2-phenylindole (DAPI), and the tissue was embedded in Vectashield Plus (Vector Laboratories, CA, USA). TMAs were scanned using a high-throughput slide scanner (Axioscan 7, Zeiss, Oberkochen, Germany).

### 2.5. Quantification of Inflammatory Cells in the APBI and BBCC Study Using Two-Color Immunohistochemistry Staining

Cell density was quantified using image-processing software (Biomas, Version 4.1 07/2018, Erlangen, Germany). The stromal compartment and the intraepithelial compartment were marked, and the corresponding areas were registered. A total tissue area of 2.7 mm^2^ (±0.88) in the APBI study and 1.1 mm^2^ (±0.46) in the BBCC study was analyzed for the infiltrating macrophages per patient. The evaluation was always blinded, and the individual spots were coded and only assigned to the corresponding patients at the end. The size, morphology, and color of the stained macrophages were used as inclusion criteria. The stromal and intraepithelial cell densities were calculated separately. The quantification of inflammatory cell densities was performed semi-automatically using image-processing software (Biomas, Erlangen, Germany). Analysis was performed using image-analysis software COUNT (Biomas Software, version 3.3; MSAB, Erlangen, Germany). The cutoff values were determined using X-tile software (version 3.6.1, Yale University, New Haven, CT, USA), where favorable and unfavorable forecasts were separated automatically.

### 2.6. Quantification of Inflammatory Cells in the APBI Study Using Four-Color Fluorescence Staining

First, the images were processed using the microscopy software Zen Blue Edition (V2.0 de/07-2017, Carl Zeiss Microscopy, Zeiss, Jena, Germany). The image settings, including color adjustments and background correction, were optimized, and any artifacts were removed. Four images were then processed, each corresponding to one of the four stains used to highlight specific markers: CD68, CD163, CD206, or MPO on the cells. The cells were identified using DAPI staining, which binds to DNA and highlights the cell’s nuclei. Tumor-infiltrating inflammatory cells (TIICs) and their density were also calculated using semiautomated image processing with the software Biomas (Biomas, Erlangen, Germany), as previously described. The first step of the analysis involved image batching, which combined two of the four previously processed images. Image 1 included CD68+ (green) and CD206+ (red) staining, while Image 2 contained MPO+ (green) and CD163+ (red) staining, with both images also showing DAPI staining. This image-batching process enabled the calculation and evaluation of TIICs. Using the image-processing software (Biomas, Erlangen, Germany), the tissue samples were then segmented into stromal and intraepithelial compartments, and the cell density was separately calculated for each compartment.

### 2.7. Missing Cell Densities from Patients

However, cell density data could sometimes only be captured in one compartment. This resulted in fewer available datasets than the total number of patients for certain markers. Most of these discrepancies can be explained by samples consisting only of stromal or epithelial tissue. Loss of material during the staining process was another factor contributing to this discrepancy. TMAs from normal breast tissue sometimes contained very little tissue or mostly fat, resulting in fewer datasets, especially intraepithelial. Spots lost during the staining or processing stages were not accounted for and were missing from the evaluation.

### 2.8. M1/M2 Shift Analysis

An M1 shift indicates that in stromal or epithelial tissue, M1 levels (CD68+CD163−) are high while M2 levels (CD68+CD163+) are low, whereas an M2 shift signifies the opposite—M1 is low and M2 is high. The remaining two combinations are classified as “non-shifted”.

### 2.9. Epithelial–Stromal Match Analysis

To our knowledge, this was the first time that an epithelial–stromal match analysis had been used in this way. In this epithelial–stromal match analysis, 16 possible groups were created by combining all possible low or high levels of M1 and M2 macrophages within the epithelial and stromal compartments. The cut-off values categorize the variables into low (0) and high (1), and each were determined with X-Tile (version 3.6.1, Yale University, New Haven, CT, USA). For example, the coding system “1010” represents a specific combination: high M1 macrophages in the epithelium, low M2 macrophages in the epithelium, high M1 macrophages in the stroma, and low M2 macrophages in the stroma. The individual risks associated with these 16 groups were then consolidated into three or four distinct risk categories. These were then grouped into low-, medium-, and high-risk categories. In the BBCC group, they were also categorized as early-risk.

### 2.10. Statistical Analysis

Kaplan–Meier plots and the log-rank test were used for the description and comparison of DFS rates between groups. Receiver operating characteristic curve analysis and X-tile software (version 3.6.1, Yale University, New Haven, CT, USA) were used to determine optimized cut-off values for prognostic groups. To compare the means of two or more groups, Student’s *t*-test and one-way analysis of variance (ANOVA) were used. Chi-squared test and Fisher’s exact test were used to compare the frequency distributions of categorical variables in contingency tables. Statistical analysis was performed using SPSS (Version26, IBM, Chicago, IL, USA) and Microsoft Excel (Version16, Microsoft, Redmond, Washington, DC, USA).

## 3. Results

### 3.1. Two Early-Stage Breast Cancer Cohorts

**Figure 1:**
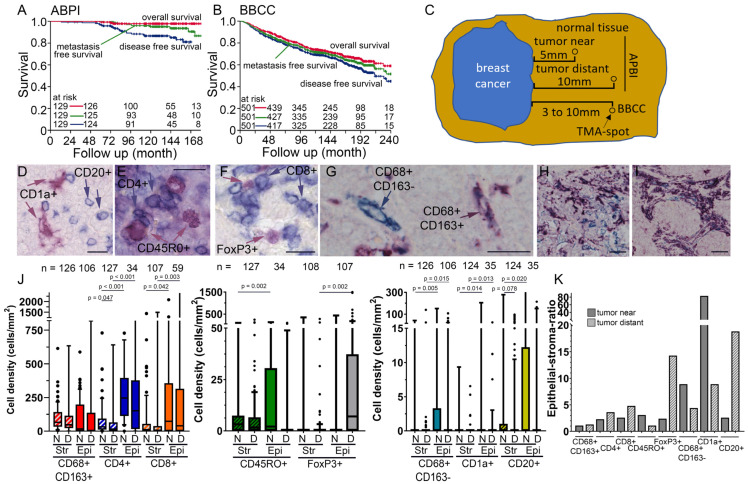
(**A**) Overall survival, metastasis-free survival, and disease-free survival of the (**A**) Accelerated Partial Breast Irradiation Study cohort and (**B**) BBCC cohort analyzed using the Kaplan–Meier method and log-rank test. (**C**) A scheme for the distance between the punched holes in the tumor near and distant normal tissue of the APBI study and the BBCC study. (**D**) Examples of inflammatory cells studied, each as double-staining (**D**) CD20+ B cells and CD1a+ dendritic cells, (**E**) CD4+ T helper cells and CD45RO+ memory T cells, (**F**) CD8+ cytotoxic T cells and FoxP3+ regulatory T cells, and (**G**) CD68+CD163− M1 macrophages and CD68+CD163+ M2 macrophages. (**H**) Example of relatively equal abundant CD68+CD163− (blue) and CD68+CD163+ (purple) macrophages. (**I**) This is an example of an extremely high frequency of CD68+CD163− (blue) and CD68+CD163+ (purple) macrophages in the stromal compartment, as well as the exclusion of macrophages in the epithelial compartment. (**J**) Stromal and intraepithelial cell density distributions of the different inflammatory cells in the tumor near and distant tissue are shown in box plots. Each subpopulation of inflammatory cells has its own color, and the stromal compartment is additionally marked with diagonal stripes. The central line represents the median values, while the box indicates the interquartile range (IQR). Whiskers represent 1.5 IQR or minimum/maximum. Outliers are indicated by points. Student’s *t*-test was used to compare the groups. (**K**) Epithelial–stromal ratio of the rates of the different inflammatory cells in the tumor near and distant tissues. N = tumor near, D = tumor distant, Str = stromal compartment, Epi = epithelial compartment; (**D**–**G**) scale bars indicate 20 µm.

Two cohorts of 129 and 501 patients with mainly early-stage breast cancer for whom tumor-related normal tissue was available were studied. The APBI cohort, which predominantly consisted of T1 tumors (95.6%) was treated with surgery followed by partial breast irradiation with interstitial brachytherapy. Median follow-up was 11 years, 10-year overall survival was 97.8%, metastasis-free survival was 95.0%, and disease-free survival (DFS) was 86.6% (Figure 1A). For the BBCC study, follow-up was 11.5 years, 10-year overall survival was 73.3%, metastasis-free survival was 80.1%, and DFS was 67.4% (Figure 1B). In the APBI cohort, we studied TIICs in tumor-near and tumor-distant normal tissues, whereas in the BBCC cohort, we studied normal tissues between 3 and 10 mm distant to the tumor (Figure 1C).

### 3.2. TIIC Densities in Normal Tissue Samples

Tissue samples from the patients were available as tissue microarrays (Supplementary Figure S1). In the APBI study, four double-stains were used to identify B cells and dendritic cells with the markers CD20 and CD1a (Figure 1D), helper T cells and memory T cells with CD4 and CD45RO (Figure 1E), cytotoxic T cells and regulatory T cells with CD8 and FoxP3 (Figure 1F), and macrophages with a double-stain for CD68 and CD163 (Figure 1G). Macrophages stained for CD68 alone were interpreted as having a more M1 phenotype, and macrophages stained for CD68 and CD163 were interpreted as having a M2-like phenotype. There were extreme differences in the frequency of stromal and epithelial inflammatory cells in normal tissues. The most common cells in the stromal compartment were CD68+CD163+ double-positive cells and CD4+ and CD8+ T cells (Figure 1H). CD4+ and CD8+ T cells were most abundant in the epithelial compartment, followed by CD68+CD163+ double-positive macrophages. Memory T cells and regulatory T cells were less frequent, and the lowest infiltrations were by CD68+CD163− TAMs, dendritic cells, and B cells. Among all inflammatory cells, intraepithelial cell densities were higher than those in the stromal compartment. The ratio of intraepithelial frequencies of inflammatory cells to stromal cells was quite similar in tumor-near and -distant tissues (Figure 1I). There were greater differences only in the inflammatory cells with very low densities.

### 3.3. M1 and M2 Macrophages in the APBI Study

**Figure 2:**
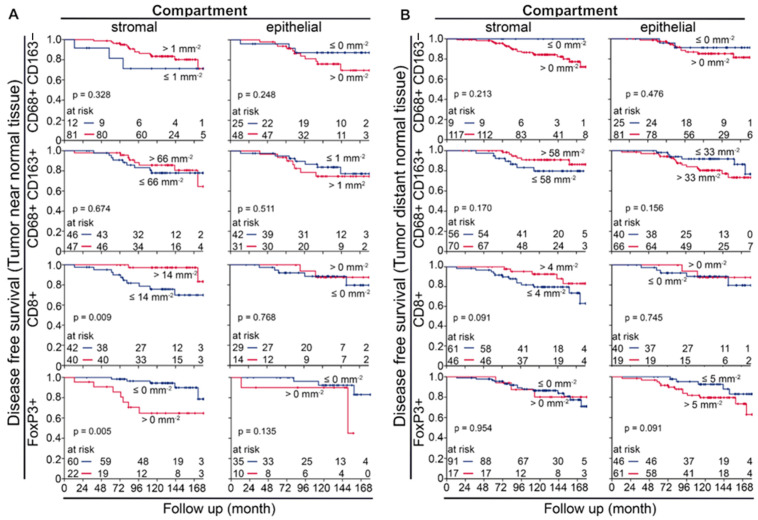
Kaplan–Meier plots of disease-free survival calculated with immune-infiltrating inflammatory cells in the stromal and intraepithelial compartments of (**A**) a tumor near normal tissue and (**B**) a tumor distant from normal tissue from tumor samples from the APBI early breast cancer tissue study. CD68+ and CD163− are considered cytotoxic M1 macrophages, CD68+ and CD163+ are considered immunosuppressive M2 macrophages, CD8+ are cytotoxic T cells, and FoxP3+ are considered immunosuppressive regulatory T cells.

We investigated the prognostic significance of measured cell densities regarding DFS in Kaplan–Meier plots with a special focus on TAMs. In normal tissues near and distant from the tumor, there were mostly weak associations between densities of measured TIICs and DFS (Figure 2 and Supplementary Figure S2). However, in both of the localizations studied, there was some tendency for CD68+CD163− TAMs in the stromal compartment of the tumors near normal tissue to be a favorable prognostic factor and in other compartments to be unfavorable. Few CD68+ CD163+ in the epithelium and many in the stroma tended to be favorable (Figure 2A,B). There were no relevant correlations with prognosis for CD4+, CD45RO+, CD1a+, and CD20+ TIIC densities (Supplementary Figure S2). High stromal CD8+ (*p* = 0.009) and low FoxP3+ (*p* = 0.005) cells in the tumors near normal tissue were associated with a significantly improved DFS (Figure 2A). Within the epithelial compartment, these inflammatory cells had hardly any prognostic significance (Figure 2A,B).

**Figure 3:**
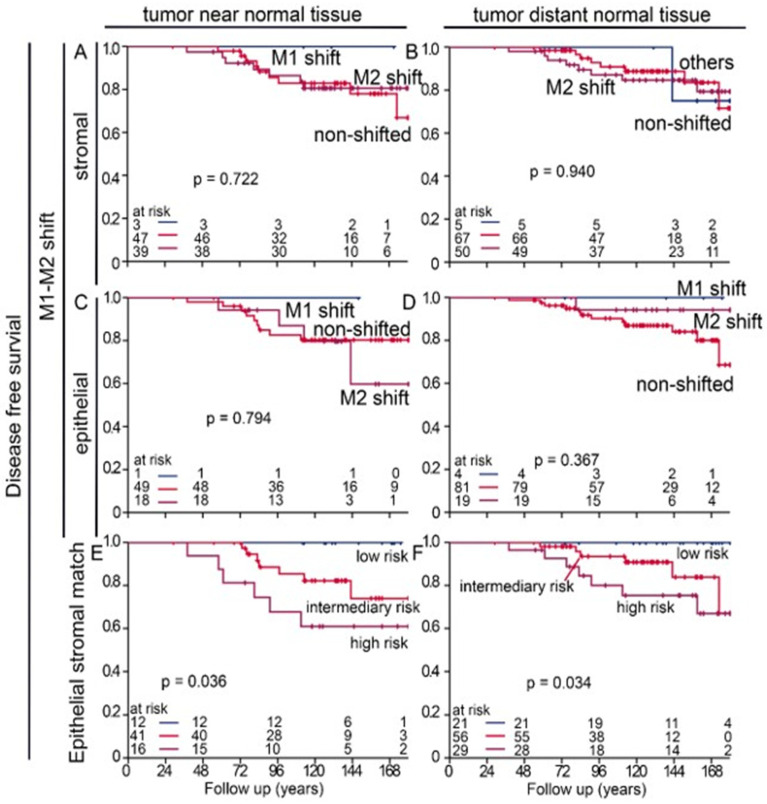
Risk analysis by M1/M2 macrophage shift and epithelial–stromal match analysis in the APBI early breast cancer cohort. (**A**–**D**) Disease-free survival calculated with Kaplan–Meier plots of specific cell densities of macrophage shifts in either the (**A**,**B**) stromal or (**C**,**D**) epithelial compartment of (**A**,**C**) a tumor near normal tissue and (**B**,**D**) a tumor distant from normal tissue and their impact on DFS. M1 shift is defined as CD68+CD163− M1 rates are high, and CD68+CD163+ M2 rates are low. M2 shift is reversed accordingly. The remaining two combinations are classified as “non-shifted”. (**E**,**F**) Epithelial–stromal match analysis with risk assessment and its impact on the DFS of tumor samples from (**E**) a tumor near normal tissue and (**F**) a tumor distant from normal tissue. In this epithelial–stromal match analysis, 16 possible groups were created by combining all possible low (0) or high (1) levels of M1 and M2 macrophages within the epithelial and stromal compartments and then were summarized into three risk groups. The individual groups are shown in Supplementary Figure S3, and Table 2 lists the groups summarized as low risk, intermediate risk, and high risk.

As TAMs had a certain prognostic importance and can be studied within the context of the two polarization groups of the M1 or M2 shift, we carried out further analyses for these subgroups. While only very few samples expressed an M1 shift, it was very prognostically favorable in both tumor-near and -distant normal tissue and in the stromal and epithelial compartments. In contrast, DFS was significantly worse in patients whose normal tissue was classified as M2-shifted or non-shifted (Figure 3A–D). It is important to mention that non-shifted cells could reflect a certain state; a study by Xue et al. suggests that macrophages show transitional stages, which depend on environmental factors [21]. We also studied the ratio of many or few macrophages of the two polarization groups in the stromal and intraepithelial compartments. We called this an epithelial–stromal match analysis. All individual combinations of low or high densities of CD68+CD163− and CD68+CD163+ macrophages in the epithelial and stromal compartments were tested for their risk and then were summarized into three groups (Supplementary Figure S3). We were able to identify the groups that had extremely good DFS without any events and groups that had extremely poor survival with very early events. The largest groups were patients with an intermediate risk (Figure 3E,F).

### 3.4. M1 and M2 Macrophages in the BBCC Study

**Figure 4:**
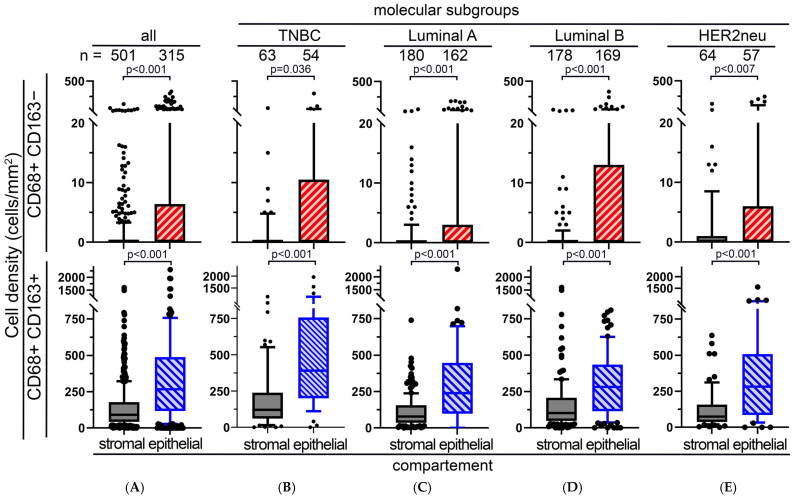
M1 and M2 rates in the BBCC cohort. Box plots of stromal and intraepithelial cell density distributions of peri-tumoral normal tissue of CD68+CD163− and CD68+CD163+ macrophages in (**A**) the total cohort, (**B**) the triple-negative, (**C**) Luminal A, (**D**) Luminal B, and (**E**) HER2neu molecular subgroups of tissue samples from the BBCC study. The central line within the box represents the median value; the box itself extends from the 25th to the 75th percentile. Whiskers are drawn from the 10th percentile to the 90th percentile. Values above and below are indicated by individual points. The value “n” indicates the total amount of values for each location. Student’s *t*-test was used to compare the groups.

**Figure 5:**
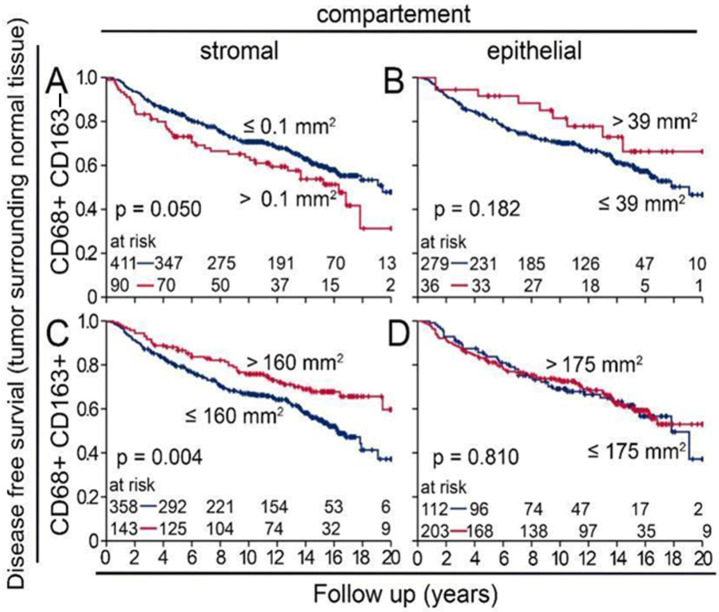
Kaplan–Meier plots of disease-free survival of specific macrophage cell densities in the (**A**,**C**) stromal and (**B**,**D**) intraepithelial compartments of peri-tumoral normal tissue from the BBCC study. CD68+ and CD163− are considered cytotoxic M1 macrophages, CD68+ and CD163+ are considered immunosuppressive M2 macrophages, CD8+ are cytotoxic T cells, and FoxP3+ are considered immunosuppressive regulatory T cells.

**Figure 6:**
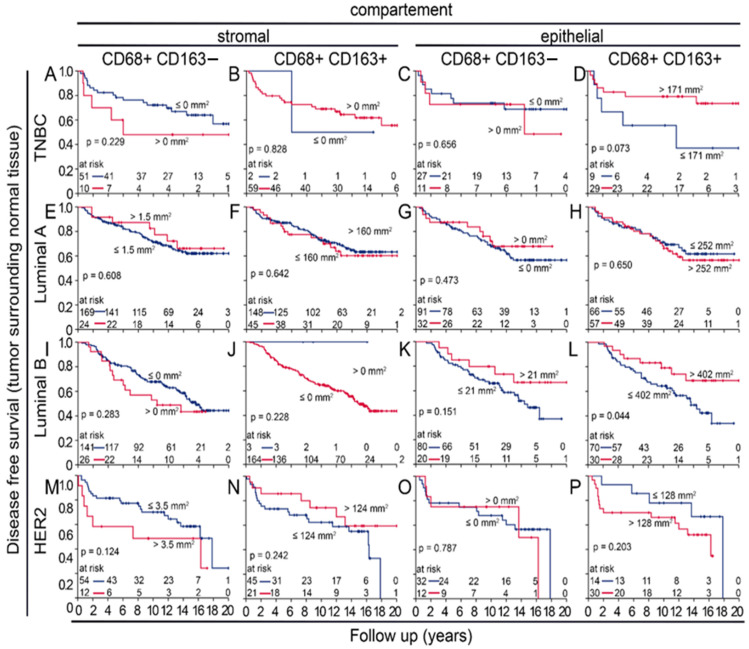
(**A**–**P**) Kaplan–Meier plots of disease-free survival of specific macrophage cell densities in the stromal and intraepithelial compartment of peri-tumoral normal tissue from the BBCC study in (**A**–**D**) TNBC, (**E**–**H**) Luminal A, (**I**–**L**) Luminal B, and (**M**–**P**) HER2 subtypes of breast cancer. CD68+ and CD163− are considered cytotoxic M1 macrophages, CD68+ and CD163+ are considered immunosuppressive M2 macrophages, CD8+ are cytotoxic T cells, and FoxP3+ are considered immunosuppressive regulatory T cells.

**Figure 7:**
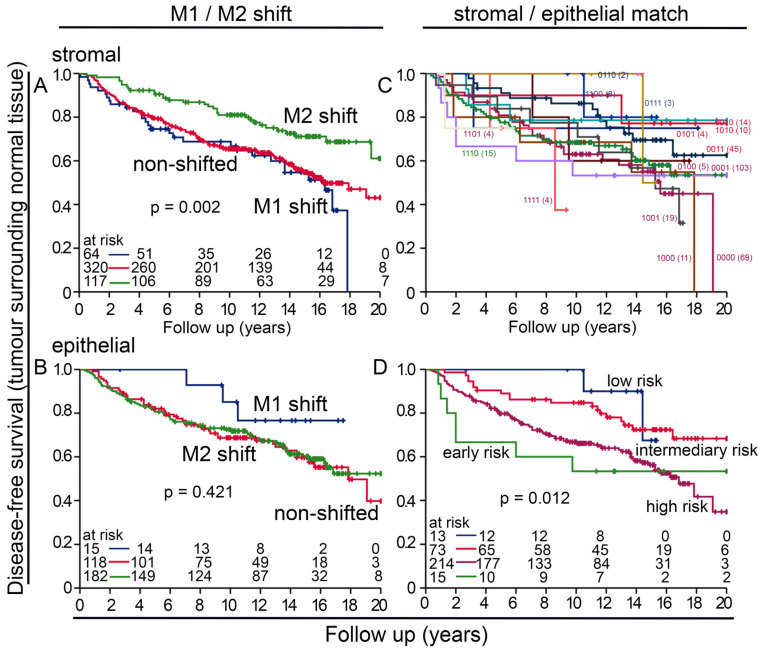
Risk analysis by M1/M2 macrophage shift and epithelial–stromal match analysis in the BBCC early breast cancer cohort. (**A**–**D**) Kaplan–Meier plots of disease-free survival, (**A**,**B**) specific cell densities of macrophage shifts in either the (**A**) stromal or (**B**) epithelial compartment and their impact on DFS. M1 shift is defined as follows: CD68+CD163− M1 rates are high and CD68+CD163+ M2 rates are low. M2 shift is reversed accordingly. The remaining two combinations are classified as “non-shifted”. (**C**,**D**) Epithelial–stromal match analysis with risk assessment and their impact on DFS of tumor samples from the BBCC study. In total, 16 possible groups were created by combining all possible low (0) or high (1) levels of M1 and M2 macrophages within the epithelial and stromal compartments and then were summarized into three risk groups. The four digits mean the following: first position: epithelial CD68+CD163− (M1); second position: epithelial CD68+CD163+ (M2); third position: stromal CD68+CD163− (M1); fourth position: stromal CD68+CD163+ (M2); 1 = valid; 0 = invalid. (**C**) The resulting groups by combining all possible low or high concentrations of M1 and M2 macrophages in the epithelial and stromal compartments. (**D**) The risk groups from (**C**) summarized in four risk groups.

Similarly to the APBI cohort, in the BBCC cohort, CD68+CD163− macrophages were also rare and were significantly less abundant in the stromal compartment than in the epithelial compartment of normal tissue samples (*p* < 0.001; Figure 4A). This was true for the molecular subgroups TNBC, luminal A and B, and HER2neu (*p* < 0.001; Figure 4B–E). CD68+CD163+ macrophages were significantly more frequent than CD68+ CD163− macrophages (*p* < 0.001; Figure 4A), and there were significantly more macrophages in the epithelial compartment than in the stromal compartment (*p* < 0.001; Figure 4B–E). Contrary to our results from the tumor samples, a low density of stromal CD68+CD163− macrophages in normal tissue samples was prognostically favorable (*p* = 0.050; Figure 5A), while the opposite was true for CD68+ CD163+ (*p* = 0.004; Figure 4C), where a high density was favorable. In the epithelial compartment, neither macrophage type had prognostic significance (Figure 5B,D). After grouping by molecular subtypes, high CD68+CD163− and CD68+CD163+ TAM densities were positively associated with a favorable DFS only in the luminal B group (Figure 6L). There were no clear differences between the high and low groups for TNBC (Figure 6A–D), luminal A (Figure 6E–H), luminal B (Figure 6I–K), and HER2 (Figure 6M–P). The M2 shift in the stromal compartment and the M1 shift in the epithelial compartment were associated with a favorable DFS. In the BBCC cohort, we also studied different risk groups according to M1 and M2 occurrence in the epithelial and stromal compartments (Figure 7A–D). In this analysis, we were also able to identify distinct risk groups, including a subset of patients characterized by extremely early recurrence or metastasis and poor DFS (*p* = 0.012; Figure 7C). We compared the two tumor near normal and tumor distant normal cohorts of the APBI group with the BBCC cohort. There was a high correlation between the two APBI groups. Out of the three tumors near low-risk signatures, two were also in the distant group, and, out of the five intermediate signatures, four were also in the distant group. The association of the APBI groups with BBCC was much weaker (Table 2).

### 3.5. Four Color Immunostaining of CD68, CD163, MPO and CD206 in the APBI Study

**Figure 8:**
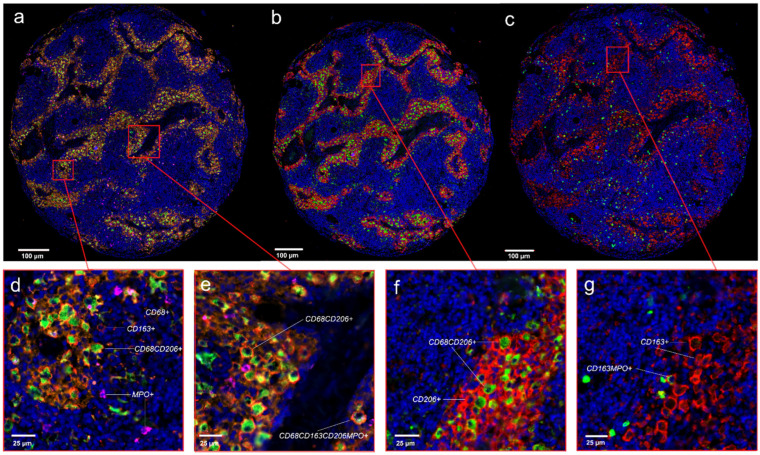
Four-color fluorescent images of breast cancer tissue microarray samples from the APBI study. (**a**–**g**) Cell nuclei are stained blue (DAPI); (**a**) in the initial image, created CD68+ cells are stained green, CD206+ cells are stained orange, CD163+ are colored in red, and MPO+ cells are colored in pink. Since cells were counted in a two-step automated process, image (**a**) was broken down into two images (**b**,**c**). (**b**,**f**) contains only CD68+ cells, stained in green, and CD206+ cells, stained in red. (**c**,**e**) consists of only CD163+ cells, colored in red, and MPO+ cells, colored in green. Scale bars in (**a**–**c**) are 100 µm; scale bars in (**d**–**g**) are 25 µm.

**Figure 9:**
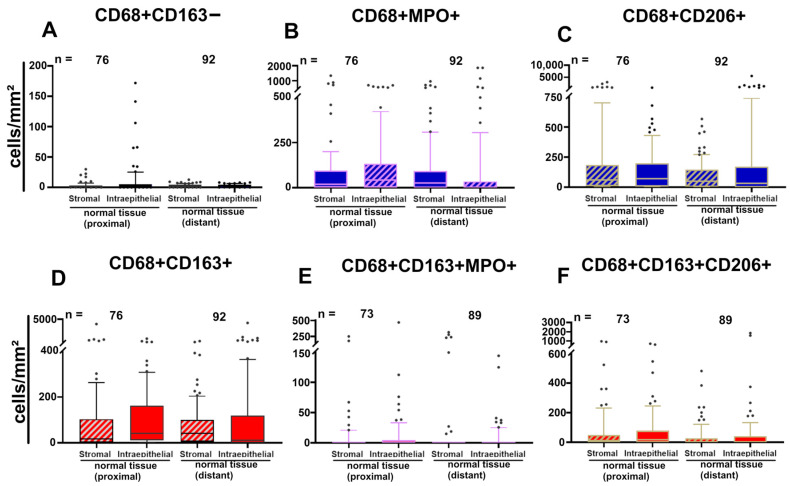
(**A**–**F**) Box plots of stromal and intraepithelial cell density distributions of tumors near normal tissue and tumors distant from normal tissue counted by the four-color fluorescence-stained tissues of the APBI study. Each subpopulation of inflammatory cells has its own color, and the stromal compartment is additionally marked with diagonal stripes. The central line within the box represents the median value; the box itself extends from the 25th to the 75th percentile. Whiskers are drawn from the 10th percentile to the 90th percentile. Values above and below are indicated by individual points. The value “n” indicates the total amount of values for each location. Student’s *t*-test was used to compare the groups; no differences were found.

To further characterize the spectrum of cytotoxic and immunosuppressive cells, the tissue sections from the APBI study were subjected to fourfold staining. In addition to anti-CD68 and anti-CD163, anti-MPO and anti-CD206 were stained using four-color fluorescence staining (Figure 8a–g). The number of macrophages per area was very similar in the tumors near and tumors distant to normal tissues. The proportion of M1-like CD68+CD163− was low, with mean values in the tumors near normal tissue in the stromal compartment of 3.0 cells/mm^2^ (SD: 5.2 cells/mm^2^), 2.7 cells/mm^2^ (SD: 2.3 cells/mm^2^) in the epithelial compartment, and similar numbers in the stromal compartment. The proportion of CD68+MPO+-expressing macrophages was significantly higher, with mean values in the tumors near normal tissue of the stromal compartment showed mean values of 98 cells/mm^2^ (SD: 223.0 cells/mm^2^) and 126.0 cells/mm^2^ (SD: 183.0 cells/mm^2^) in the epithelial compartment, and the tumors distant from normal tissue showed mean values of 97.0 cells/mm^2^ (SD: 176.0 cells/mm^2^) in the stromal compartment and 122.0 cells/mm^2^ (SD: 399.0 cells/mm^2^) in the epithelial compartment. The combination of CD68+ and the immunosuppressive marker CD206+ was also frequent, with mean values of 225.0 cells/mm^2^ (SD: 491.0 cells/mm^2^) in the stromal compartment of the tumors near normal tissue and 96.0 cells/m^2^ (SD: 123.0 cells/mm^2^) in the stromal compartment of the tumors distant from normal tissue. Suppressive CD68+CD163+ macrophages in the tumor near normal tissue were relatively abundant. These cells, combined with MPO, were very rare, and, again, were frequent when combined with CD206+ (Figure 9).

### 3.6. Prognostic Value of Macrophages in Tumor near and Distant Tissues

**Figure 10:**
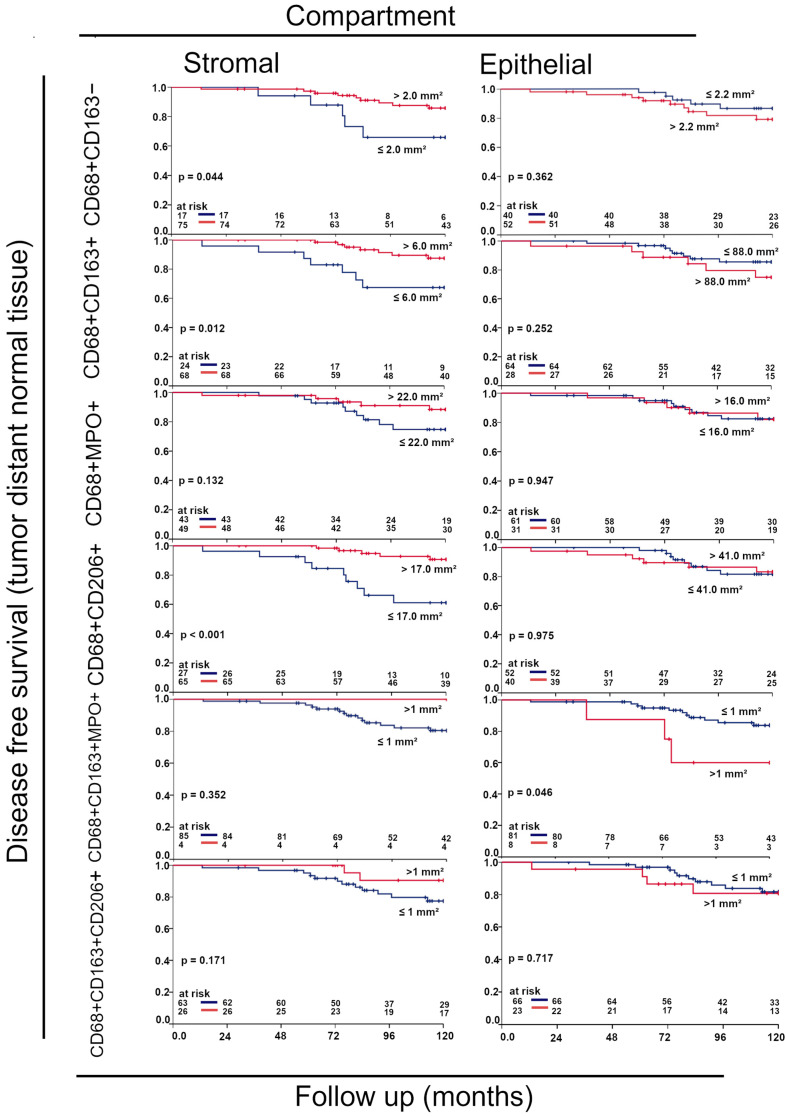
Kaplan–Meier plots of DFS of tumor distant normal tissue counted by the four-color fluorescence-stained tissues of the APBI study. CD68+ are all macrophages, CD68+ and CD163+ are considered immunosuppressive M2 macrophages, CD68+ and MPO+ are considered cytotoxic, CD68+ and CD206+ are considered more immunosuppressive cells, CD68+, CD163+, and MPO+ are not clear which cell type they are, and CD68+, CD163+, and CD206+ are immunosuppressive cells.

High rates of CD68+CD163− (*p* = 0.044), CD68+CD163+, and CD68+CD206+ macrophages (*p* < 0.001) were significantly favorable for 10-year DFS only in the stromal compartment of tumor-distant normal tissue (Figure 10). In the tumor near normal tissue, no significant results were found (Supplementary Figure S4).

### 3.7. Uni- and Multivariate Analysws of Four-Color Macrophage Staining

Using univariate and multivariate analyses, we tested three significant combinations of the four-color staining of macrophages in Kaplan–Meier graphs to determine independence. High CD68+CD206+ expression was significantly associated with reduced disease-free survival (DFS) (hazard ratio [HR]: 0.137, 95% confidence interval [CI]: 0.044–0.426, *p* < 0.001). This effect remained statistically robust after adjusting for chemotherapy, which was also independently associated with an increased risk (HR = 12.15, *p* < 0.001) (see Table 3). For CD68+/CD163+, infiltration was associated with improved outcomes (HR = 0.26, 95% CI: 0.087–0.754, *p* = 0.013), indicating a reduction in recurrence risk compared to patients with low expression. Chemotherapy remained an independent predictor of increased risk (HR = 8.21, 95% CI: 2.10–32.14, *p* = 0.003). Tumor grade tended toward significance (HR = 4.17, *p* = 0.174), but the confidence interval was wide. In the final Cox regression model incorporating CD68+, CD163−, and chemotherapy, CD68+CD163− was independently associated with a reduced risk of recurrence (HR = 0.272, 95% CI: 0.088–0.838, *p* = 0.023).

## 4. Discussion

Over the previous decades, the importance of tumor microenvironments, with their inhomogeneous composition of tumor cells, connective tissue, blood vessels, extracellular matrix, and inflammatory cells of various types, has been recognized [22]. This study complements our understanding of the tumor microenvironment in breast cancer in that we found that the surrounding normal tissue is also influenced by the tumor beyond the classical tumor boundaries and vice versa. This analysis reveals the relevance of inflammatory cell infiltration in peri-tumoral normal tissue on DFS of breast cancer patients. This distinguishes our study from previous research, which primarily focused on immune cell infiltration of the tumor itself. We found that cytotoxic T cells (CD8+), regulatory T cells (FoxP3+) and macrophages have some prognostic relevance in the tumor near normal tissue and focused further on the presence of macrophages. Prior studies have shown that CD68+CD163+ macrophage infiltration, as well as CD68+CD163− macrophage presence in the TME, are associated with reduced overall survival in breast cancer patients [23].

Previous studies on breast cancer use the anti-CD68 pan-macrophage marker, which has been shown to be mostly associated with unfavorable tumor stages and prognosis. This may be explained by the predominance of CD68-labeled macrophages being of the M2 subtype, which are typically associated with unfavorable characteristics [23]. Similar results were observed with the CD163+ marker, which also identifies M2-type macrophages and is commonly linked to advanced tumor stage and poor prognosis [23].

Therefore, the cytotoxic or immunosuppressive properties of TAMs cannot be determined using only individual markers for macrophages. As mentioned above, overall survival and DFS also depend on the phenotype of the macrophages present in the tumor itself. For example, Dai et al. found that there is a difference in tumor progression in breast cancer depending on the abundance of M1-type and M2-type macrophages [24]. CD68+CD163+ cells are classified as M2-type macrophages, which are known to promote tissue repair, angiogenesis, and reduce inflammation [25]. Other studies have also found that M2-type macrophages in the tumor environment itself are more likely to be associated with poor overall survival, as suggested by Liu et al. [26]. Furthermore, a meta-analysis by Ni et al. showed that CD68+CD163+ macrophages in the tumor microenvironment itself were associated with poor outcomes [25]. However, these studies only examined the TME, not the adjacent normal tissue. This motivated us to further distinguish between different macrophage phenotypes. For this reason, we performed two-color antigen staining using anti-CD68 and anti-CD163 antibodies, as well as four-color (4C) staining using anti-CD68, anti-CD163, anti-CD206, and anti-MPO antibodies.

As expected, the overall number of CD68+CD163− M1-type macrophages were rather low, with 0.9 cells/mm^2^ tumor near and 1.4 cells/mm^2^ tumor distant in the stromal compartment and 8.1 cells/mm^2^ and 6.0 cells/mm^2^ in the epithelial compartment of samples of the APBI studies. In a previous study of these macrophages in central tumor tissue, higher rates were registered in the stromal compartment with 21.6 cells/mm^2^ (*p* < 0.022), albeit with quite similar amounts in the epithelial compartment with 10.8 cells/mm^2^ (*p* > 0.303) [27]. The M2-type CD68+CD163+ macrophages overall were much more frequent both in the stromal compartment of normal tissue with 98.6 cells/mm^2^ and 81.3 cells/mm^2^ and in the epithelial compartment with 106.7 cells/mm^2^ and 102.6 cells/mm^2^. M2-type macrophages in the stroma of the tumor were much higher (209.4 cells/mm^2^) (*p* < 0.016) than in the epithelium (164.6 cells/mm^2^) (*p* < 0.019). One could conclude that the epithelium is particularly affected by the tumor. However, it is noteworthy that the stromal compartment, with its composition of inflammatory cells, may be particularly influenced by the tumor. So far, the normal tissue around the tumor has been studied very little. In the ABPI study, the distances were relatively close to the tumor: 5 mm for near and 10 mm for far. In the BBCC study, distances were similar between 3 mm and 10 mm. In both studies, no tumor cells were found in normal tissue. However, inflammatory processes in the sense of field cancerization or occult ductal carcinoma in situ (DCIS) cannot be ruled out as having an influence on the tissue. A study by Reddy et al. investigating CD68+ macrophage infiltration in normal tissue surrounding inflammatory breast cancer (IBC), collected ≥ 5 cm from the primary tumor, and found an enrichment of CD68+ macrophage infiltration in normal tissue of IBC patients [28]. However, this study is limited by a relatively small sample size of initially 8 IBC patients and 60 non-IBC patients [28]. For CD68+CD163− macrophages, we could not find consistent results, as the APBI 4C cohort suggests that a high infiltration of CD68+CD163− macrophages in the stromal compartment of tumor distant normal tissues improves 10-year DFS (*p* = 0.044), whereas the BBCC study suggests that a rather low prevalence of CD68+CD163− cells correlates with a better prognosis (*p* = 0.05). There are probably different reasons why immunosuppressive M2 macrophages can also lead to a favorable prognosis. M2-like macrophages within the tumor itself can take up different roles. They are known to promote immune proliferation, angiogenesis, metastasis, immune invasion, and tissue remodeling, and show anti-inflammatory traits [29]. However, M1- and M2-like differentiation may not be entirely accurate, as macrophages are a heterogeneous group [29]. M2-like macrophages, for example, may be further divided into M2a, M2b, M2c, as suggested by Mantovani et al. [30]. M2a and M2b macrophages may play an immunomodulatory role, whereas M2c macrophages promote tissue remodeling [30]. Certain subsets of macrophages in peri-tumoral stroma may still perform tissue remodeling, but also limit inflammation or function as a barrier for tumor invasion. Another possible explanation is cytokine-driven functional specialization. Within the tumor for example, M2-like macrophages may be presented with certain cytokines, such as hypoxia-induced factor, which may promote angiogenesis within the tumor and promote tumor progression [31]. These specific cytokines may be absent in the peri-tumoral normal tissue, and M2-like macrophages may, therefore, exhibit distinctive features. Another example of the different effects of immunosuppressive cells are regulatory T-cells (Tregs). We observed FoxP3+ Tregs to be either favorable or unfavorable prognostic markers. In an inflammatory tumor milieu, Tregs were prognostic favorable, possibly due to the suppression of inflammation and the subsequent suppression of the pro-tumoral growth stimulation of inflammation. In cancers with low infiltration of inflammatory cells, Tregs are unfavorable, probably due to the suppression of anti-tumoral activity of cytotoxic inflammatory cells. Thus, M2-like macrophages may exhibit dual activity depending on inflammation or the presence of other inflammatory cells [32].

In the 4C analysis, we found that high infiltration of CD68+CD163+ macrophages correlated with improved DFS, particularly in the stromal compartment of tumor distant normal tissue. These findings were consistent across both the APBI and the BBCC study. This also undermines the positive prognostic relevance of CD68+CD163+ macrophages in the stromal compartment of normal tissue surrounding the tumor. This finding is especially remarkable since M2-like TAMs are traditionally considered a negative prognostic factor due to their immunosuppressive properties. In our investigation of the central tumor and invasive front, samples of the same APBI cohort M2-like TAMs were also clearly associated with reduced DFS [24].

To further substantiate M1-type macrophages and their cytotoxicity, we used CD68+MPO+ macrophages. In the current literature, limited data exist regarding CD68+MPO+ macrophages. Macrophages characterized by MPO expression are typically a pro-inflammatory macrophage subset with microbicidal activity [33]. A study by Ambrosone et al. indicates that MPO, released by macrophages and neutrophiles, may play a role when treating breast cancer, as MPO may contribute to treatment efficacy [34]. Our findings suggest that CD68+MPO+ cells in the stromal compartment of peri-tumoral normal tissue may affect DFS (Figure 9 C; *p* = 0.132). However, due to the lack of extensive studies on CD68+MPO+ macrophages, further research should be performed in the future.

Additionally, we aimed to analyze macrophages beyond the conventional M1/M2-type dichotomy by looking at the range of macrophage subtypes in between. While we detected CD68+CD163+MPO+ cells, they were very rare in both compartments, and only limited conclusions can be drawn from their detection. However, we found statistical relevance for DFS for CD68+CD163+MPO+ cells in the epithelial compartment of tumor distant normal tissue. If these cells exist, it is not clear whether they belong to the M1 or M2-type of TAMs. It can be speculated that they rather belong to the M2 spectrum.

To identify M2 macrophages even more reliably, we investigated both CD68+CD206+ and CD68+CD163+CD206+ macrophages. Cells expressing these markers are generally considered to be M2-type macrophages [35]. Bobrie et al. classified CD68+CD206+ macrophages as a subtype of M2 type macrophages [36]. They found these types of macrophages to improve prognosis in patients with triple-negative breast cancer (TNBC); moreover, CD206+ expression in macrophages was associated with smaller tumor size and higher TILs levels [36]. Bobrie et al. also used four markers (CD68, CD163, CD206, and IRF 8) to study the importance of macrophage infiltration in TNBC, making the study design comparable to ours [36]. Favorable prognosis of CD206+ TAM infiltration was also found in other studies [23]. Thus, it suggests that, despite their M2-like polarization, CD206+ TAMs can be associated with a favorable prognosis. We have also observed this in our results. We found that CD68+CD206+ infiltration was common and CD68+CD163+CD206+ macrophage infiltration in the stromal, as well as in the epithelial compartment was rather rare. Our results indicate that CD68+CD206+ macrophages have a significant impact on 10-year DFS. Our findings on CD68+CD163+CD206+ M2-type macrophages suggest a potential relevance of these macrophages in the stromal compartment of tumor distant normal tissue (Figure 9F; *p* = 0.171).

To better assess the importance of M1 and M2 macrophages, we performed two additional analyses. The M1/M2 shift analysis considers whether there are many or few M1-type or M2-type cells in the stromal or epithelial compartment of each patient (Figure 7A,C). Although the M1/M2 shift analysis previously identified the M2 shift group as having an extremely unfavorable DFS [24], it did not satisfactorily stratify patients by peri-tumoral normal tissue. Risk groups could be identified much better with epithelial stromal match analysis. Although the tumor-near and -distant normal tissues of the APBI study were evaluated completely independently, there was a high degree of agreement between the different risk groups. And even the BBCC group still had some agreement with the APBI cohort. However, within a risk group, it is not clear how the individual subgroups are related.

In addition to macrophages, CD8+ cytotoxic T cells and their counterparts, FoxP3+ regulatory T cells, are another very interesting group of inflammatory cells that expressed an unexpected prognostic relevance in the normal tissue surrounding the tumor. These cells and the macrophages clearly show that an environment determined by the tumor is present in the normal tissue surrounding the tumor. Since the inflammatory effect of tumor-infiltrating lymphocytes and their immunosuppressive effect in the tumor can significantly influence the prognosis, it is also possible that such mechanisms originate from the normal tissue surrounding the tumor. For example, inflammatory, and thus proliferative, stimuli could be triggered by inflammatory cells in the normal tissue. Similarly, an immunosuppressive environment could prevent the immune system from acting against the tumor. These mechanisms could also promote tumor ingrowth into normal tissue, or the opposite of all these mechanisms. The normal tissue surrounding the tumor is a structure that has not been sufficiently studied, and should be considered.

### Limitations

One clear limitation of this study is that some patients were lost during the various staining procedures because no evaluations were available from them. Evaluations using large TMAs mean that if individual spots are lost or damaged during staining, they cannot be replaced, so the data for those spots are unavailable. Patients for whom spots were unavailable were not included in the evaluation, resulting in different numbers of patients being evaluated. CD68 is known as a pan-macrophage marker, which serves as a marker for both M1-like and M2-like macrophages [37,38]. CD163 is known as a scavenger receptor, which is highly specific for M2-like macrophages [37]. Therefore, we used CD68+CD163− as a M1-like marker and CD68+CD163+ as a marker specific to M2-like macrophages, as they have served as reliable markers in previous studies, using the M1/M2 dichotomy [37,38,39,40]. Several studies have used either one or the combination of both markers to distinguish between M1-like and M2-like macrophages [37,38,40]. One clear advantage of this study is that we always used both markers for double-staining. Therefore, we can clearly distinguish between CD68+CD163− and CD68+CD163+ cells. To distinguish them even further, CD206 can be used as a macrophage marker in combination with CD68 and CD163 [39]. However, we acknowledge that limitations have to be made to the model of M1/M2 dichotomy of macrophages, especially for MPO, since it is rather known as a marker in neutrophiles [41]; however, a study by Gurski et al. suggests some relevance for MPO in some cases of macrophage subsets [33]. However, since the prerequisite for all counted subgroups of macrophages was that cells had to be CD68+, we can assume with relative certainty that we are only studying macrophages. For further studies to be made, we suggest adding iNOS [42]. For further differentiation, some studies used CD204 as a M2-like macrophage marker [43,44]. In addition to the markers used in our panel, this could further specify between M1-like and M2-like macrophages.

## 5. Conclusions

Normal tissue surrounding hormone-receptor positive breast cancer tumors is clearly influenced by the tumor and could also influence the tumor in terms of growth and metastasis. Tumor-influenced inflammatory cells in the surrounding normal tissue could prevent the immune system from acting against the tumor and promote tumor growth through inflammation. As their prognostic relevance in normal tissue is completely opposite to what was observed in tumor samples, the role and mechanisms of action of tumor-associated macrophages in normal tissue around breast cancer tumors should be studied more closely.

## Figures and Tables

**Table 1:** Clinical characteristics of the patients included in the APBI and BBCC study.

Variables	APBI Cohort (*n* = 129)	BBCC Cohort (*n* = 501)
Age (years)	59.1; <50: 25 (19.4%); >50: 104 (80.6%);	58.1; <50: 136 (27.1%); >50: 365 (72.9%);
T category	pT1mic: 6 (4.7%); pT1a: 8 (6.2%); pT1b: 31 (24%); pT1c: 75 (58.1%); pT2: 9 (7%);	pT1: 253 (50.5%); pT2: 159 (31.7%); pT3: 18 (3.6%); pT4: 33 (6.6%); n.a.: 38 (7.6%);
N category	N0: 126 (97.7%); N1: 3 (2.3%);	pN0: 360 (71.9%); pN1: 117 (23.4%); n.a.: 24 (4.8%);
M category	M0: 129 (100%); M1: 0 (0%);	M0: 479 (95.6%); M1: 22 (4.4%);
Histological typing	ductal: 91 (70.5%); lobular: 19 (14.7%); other: 19 (14.7%);	ductal: 266 (53.1%); lobular: 72 (14.4%); other: 92 (18.4%); n.a.: 71 (14.2%);
Molecular class	luminal A: 85 (65.9%); luminal B: 33 (25.6%); Her2: 6 (4.7%); n.a.: 5 (3.9%);	TNBC: 36 (7.2%); luminal A: 113 (22.6%); luminal B: 109 (21.8%); HER2: 66 (13.2%); n.a. (35.3%);
Histological grading	G1: 34 (26.4%); G2: 90 (69.8%); G3: 3 (2.3%); n.a.: 2 (1.6%);	G1: 47 (9.4%); G2: 325 (64.9%); G3: 119 (23.8%); n.a.: 10 (2%);
Estrogen receptor status	positive: 125 (96.9%); negative: 1 (0.8%); n.a.: 3 (2.3%);	positive: 53 (10.6%); negative:1: 200 (39.9%); n.a.: 248 (49.5%);
Progesterone receptor status	positive: 118 (91.5%); negative: 9 (7%); n.a.: 2 (1.6%);	positive: 71 (14.2%); negative:1: 181 (36.1%); n.a.: 249 (49.7%);
Her2 status	positive: 7 (5.4%); negative: 119 (92.2%); n.a.: 3 (2.3%);	positive: 209 (41.7%); negative: 94 (18.8%); n.a.: 198 (39.5%);
Ki67	<20%: 102 (79.1%); >20%: 24 (18.6%); n.a.: 3 (2.3%);	<20%: 166 (33.1%); >20%:1: 152 (30.3%); 2: 183 (36.5%);

n.a. = not available.

**Table 2:** Comparison of epithelial–stromal match analysis in APBI and BBCC. DFS risk assessment of macrophages in the epithelial and stromal compartments based on high (1) or low (0) levels of M1 or M2 and matches between the APBI cohort and the BBCC cohort. The four digits mean the following: first position: epithelial CD68+CD163− (M1); second position: epithelial CD68+CD163+ (M2); third position: stromal CD68+CD163− (M1); fourth position: stromal CD68+CD163+ (M2). The risk is categorized as low risk, intermediary risk, high risk, and additionally in the BBCC study as early risk.

	ABPI		BBCC
Risk	Near	Distant	
low risk			1000
	1001	1001	
	1101	1101	
	1110		
			1100
		0010	
		0110	0110
			0111
		0101	
intermediary risk	0000	0000	
	1100	1100	
	0100	0100	
	1111	1111	
	0101		0101
		1110	
			1010
			0010
			0011
high risk			0000
	0001	0001	0001
	0111		
			0100
			1000
			1001
			1101
			1111
early risk			1110

**Table 3:** Univariate and multivariate analysis of according to Cox’s proportional hazards model.

BREAST CANCER	Univariate Analysis			Multivariate Analysis		
Variable	Hazard Ratio	95% Confidence Interval	*p*-Value	Hazard Ratio	95% Confidence Interval	*p*-Value
Grade G1 [n = 23] v. G2/3 [n = 64]	2.985	0.368–24.193	0.306	3.333	0.424–26.179	0.252
Chemotherapy yes [n = 83] v. no [n = 4]	11.374	1.193–108.472	0.035	12.153	2.937–50.294	0.001
Ki67 ≤ 20% [n = 67] v. >20 [n = 20]	1.983	0.58–6.779	0.275	1.930	0.583–6.394	0.282
Stromal CD68+CD206+ ≤ 17 [n = 24] v. >17 cells/mm^2^ [n = 63]	0.121	0.034–0.428	0.001	0.137	0.044–0.426	0.001
Age ≤ 49 y [n = 15] v. >49 y [n = 72]	1.469	0.24–8.992	0.678	---	---	---
Lobular histology [n = 74] v. other [n = 13]	1.018	0.189–5.477	0.983	---	---	---
Hormone therapy: yes [n = 5] v. no [n = 82]	0.337	0.039–2.912	0.323	0.322	0.038–2.708	0.297

Stromal CD68+CD206+ represents macrophages in stromal tumor distant normal tissue.

## Data Availability

The datasets generated and/or analyzed in the present study are not publicly accessible but can be provided by the corresponding author upon reasonable request.

## Supplementary Materials

The following supporting information can be downloaded at: https://www.mdpi.com/article/10.3390/cells14110828/s1, Figure S1: Example of a TMA and TMA spots; Figure S2: Kaplan–Meier plots of disease-free survival calculated with immune infiltrating inflammatory cells in the stromal and intraepithelial compartments; Figure S3: Epithelial-stromal match of individual combinations of low or high densities of CD68+CD163− and CD68+CD163+ macrophages in the epithelial and stromal compartments; Figure S4: Kaplan–Meier plots of disease-free survival of tumor near normal tissue counted by the four-color fluorescence-stained tissues of the APBI study.

## Abbreviations

The following abbreviations are used in this manuscript:

MDPI	Multidisciplinary Digital Publishing Institute
DOAJ	Directory of open access journals
TLA	Three letter acronym
LD	Linear dichroism
HRs	Hormone receptors
HER-2	Human Epidermal Growth Factor Receptor 2
PR	Progesterone Receptor
TILs	Tumor-Infiltrating lymphocytes
TME	Tumor Microenvironment
TAMs	Tumor associated macrophages
APBI	Accelerated Partial Breast Irradiation
PDR	Pulse-dose-rate
HDR	High-dose-rate
DFS	Disease-free survival
BBCC	Bavarian Breast Cancer Cases and Controls
TMA	Tissue Microarray
MPO	Myeloperoxidase
DAPI	4′6-diamidino-2-phenylindole
TIICs	Tumor-infiltrating inflammatory cells
ANOVA	One-way analysis of variance
IQR	Interquartile Range
Str	Stromal compartment
Epi	Epithelial compartment
4C	Four-color
IBC	Inflammatory Breast cancer
TNBC	Triple-Negative Breast Cancer
HE	Hematoxylin and Eosin
