# Supplementary material for: Prognostic Significance of Macrophage Phenotypes in Peri-Tumoral Normal Tissue of Early-Stage Breast Cancer
Source: Cells. 2025 Jun 3;14(11):828. doi: 10.3390/cells14110828 (PMC12155421; doi:10.3390/cells14110828)
Supplement: Supplementary file 1 [file cells-14-00828-s001.zip › cells-3638612-supplementary.pdf]

**Supplementary material:**

Prognostic significance of macrophage phenotypes in peri-tumoral normal tissue of early-stage breast cancer.

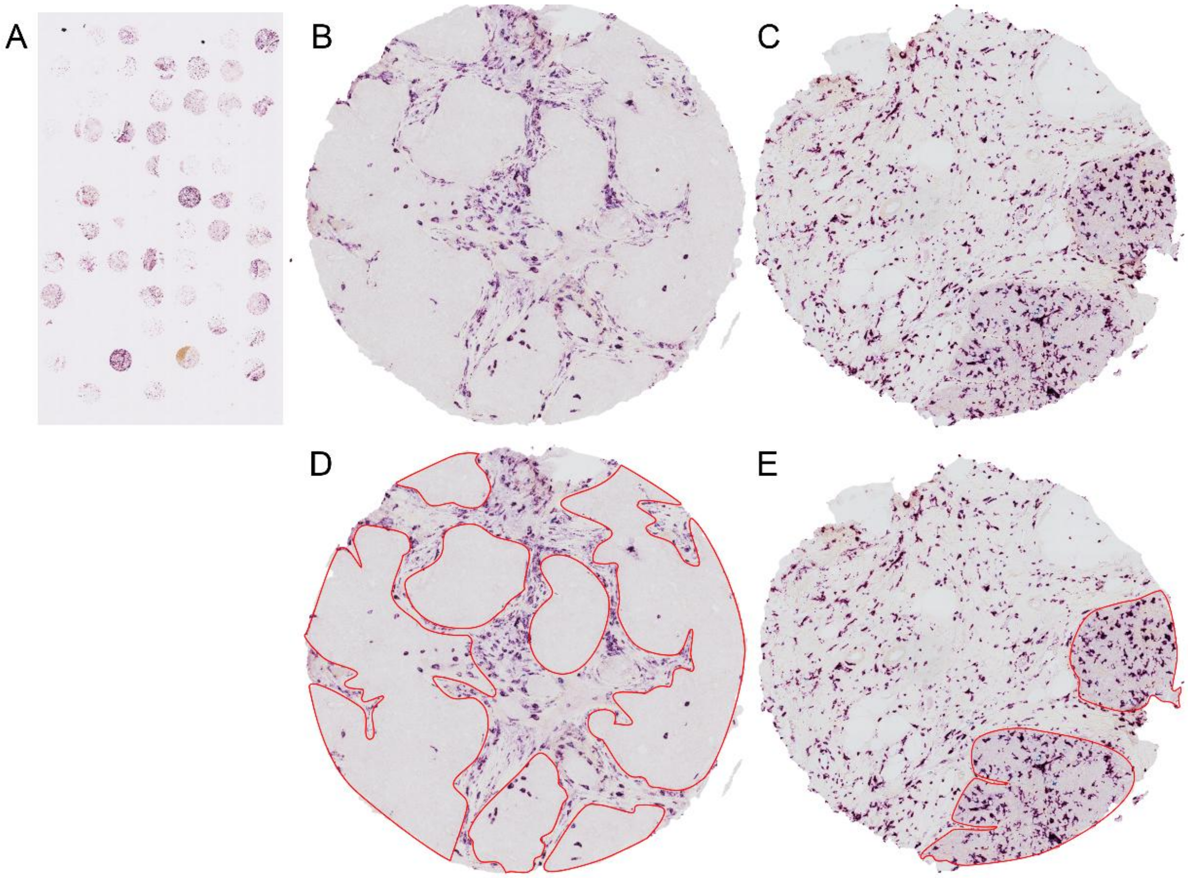


Supplementary Figure S1: Example of a TMA and TMA spots. (A) A complete tissue microarray stained with anti CD68 (blue) and anti CD163 (red) antibodies. Blue CD68+ cells remain blue while double stained cells of CD68+ and CD163+ show a purple color. (B) A spot with macrophages almost exclusively in the stromal compartment and no macrophages in the epithelial compartment. (C) A spot with a relatively equal frequency of cells in the stromal and epithelial compartments. (D) Spot B and (E) Spot C with marked areas of the epithelium. The area of the epithelium is outlined in red.

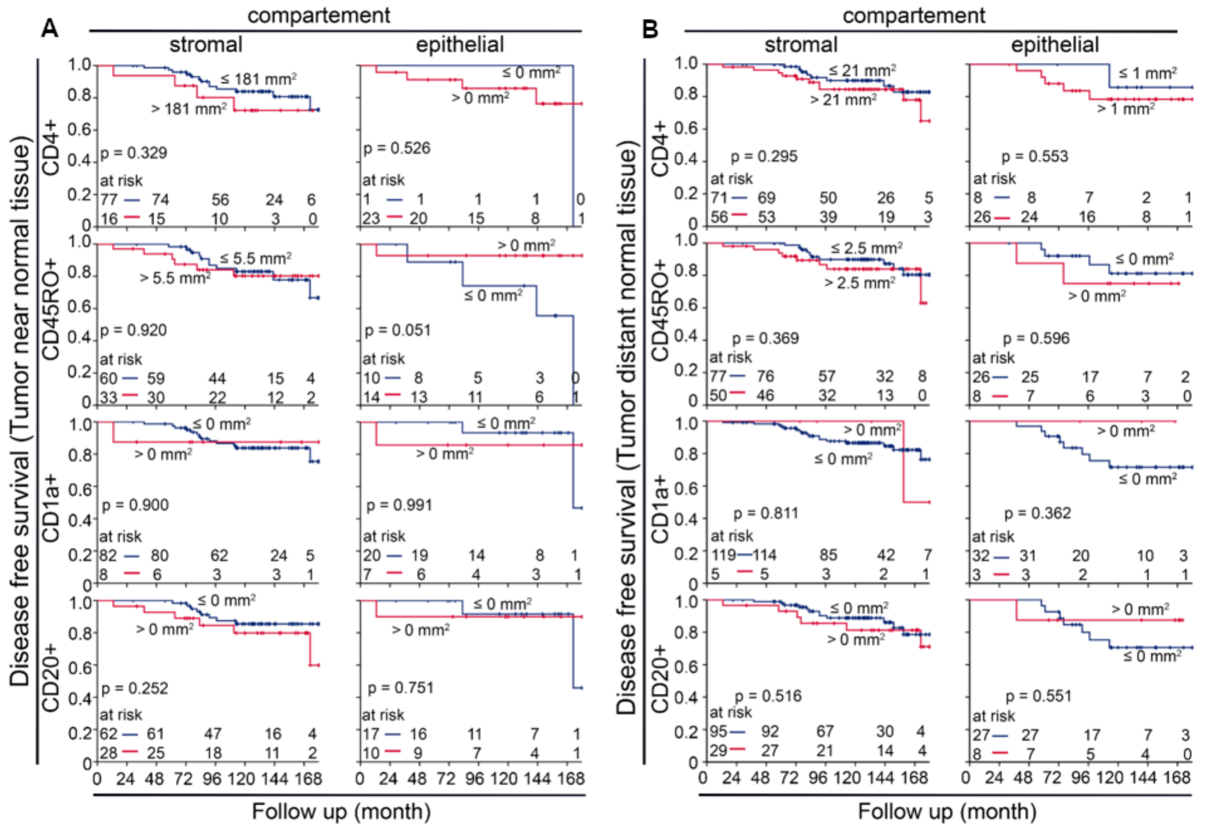


Supplementary Figure S2: Kaplan-Meier plots of disease-free survival calculated with immune infiltrating inflammatory cells in the stromal and intraepithelial compartments of (A) tumor-near normal tissue and (B) tumor distant normal tissue from tumor samples from the APBI early breast cancer tissue study. CD4+ are helper T cells, CD45RO+ are memory T cells, CD1a+ are dendritic cells and CD20+ are B cells.

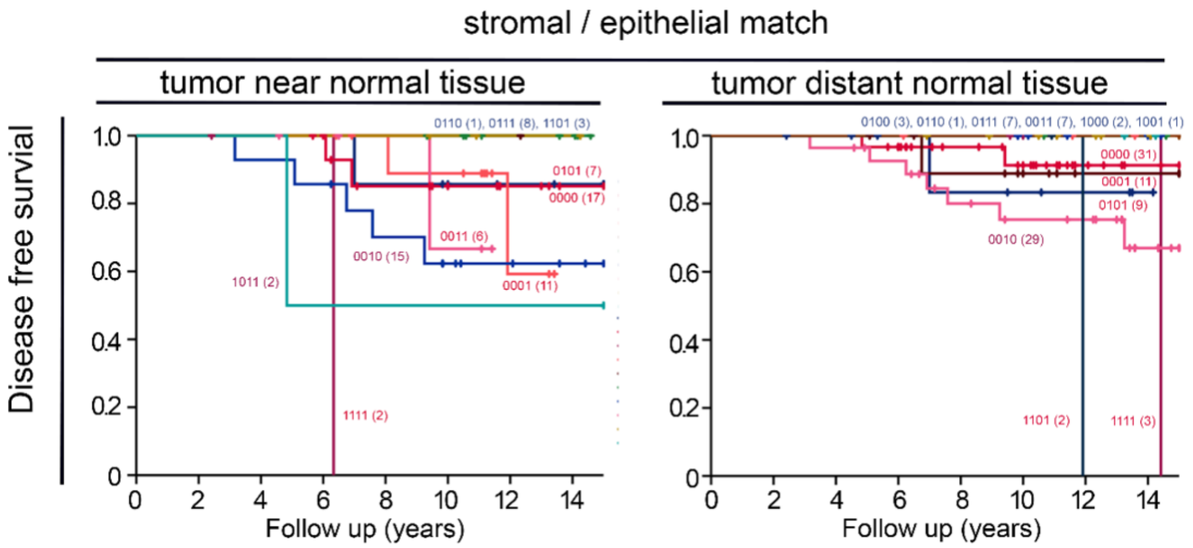


Supplementary Figure S3: Epithelial-stromal match of individual combinations of low or high densities of CD68+CD163- and CD68+CD163+ macrophages in the epithelial and stromal compartments of tumor near and tumor distant normal tissue from tumor samples of the APBI study. The risks summarized in three classes are shown in Figure 3.

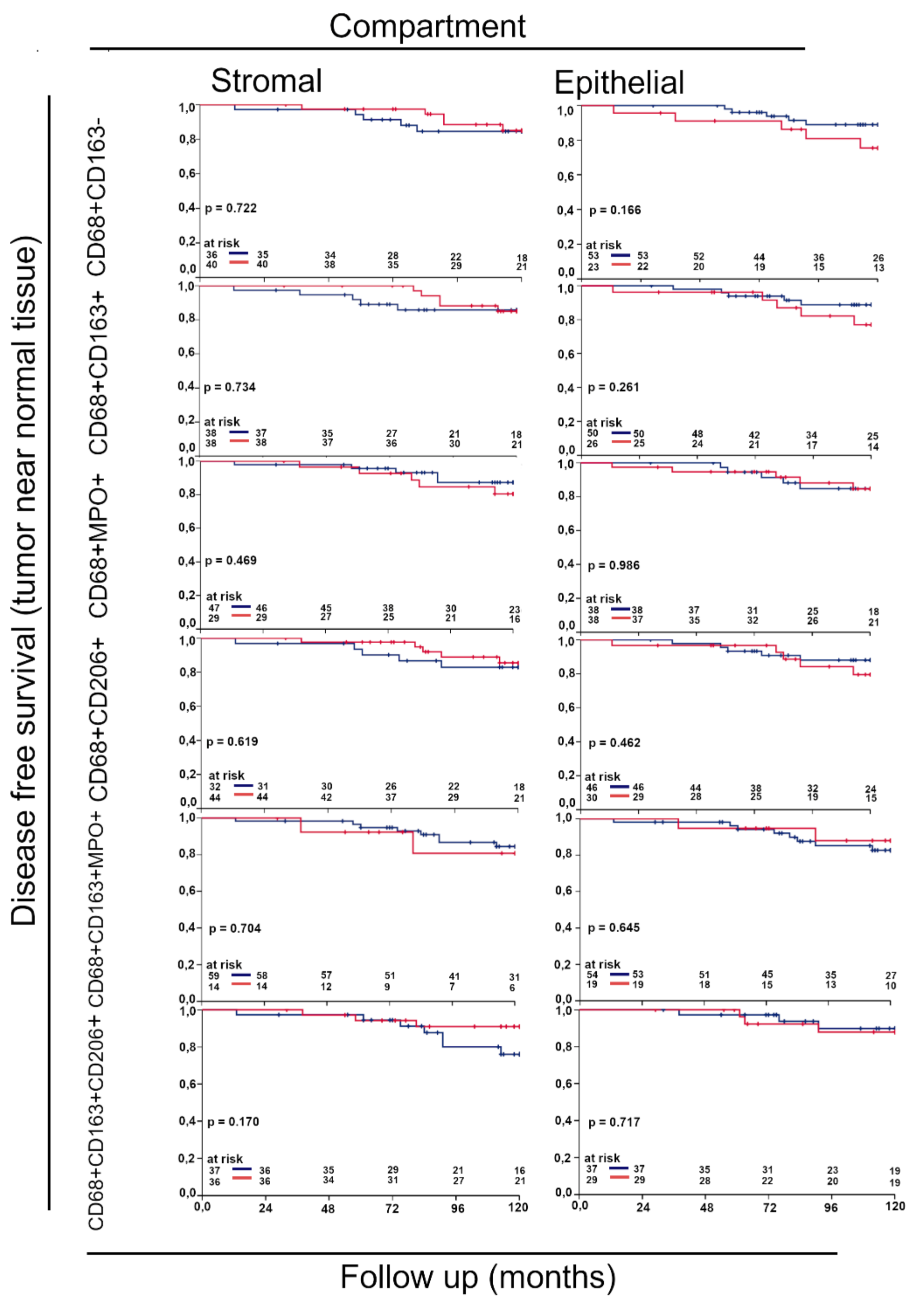


Supplementary Figure S4: Kaplan-Meier plots of disease-free survival of tumor-near normal tissue counted by the four-color fluorescence-stained tissues of the ABPI study. CD68+ are all macrophages, CD68+, CD163+ are considered immunosuppressive M2 macrophages, CD68+, MPO+ are considered cytotoxic,CD68+, CD206+ are considered more immunosuppressive cells, CD68+, CD163+, MPO+ is not clear which cell type it is, CD68+, CD163+,CD206+ are immunosuppressive cells.
